# Pogostemon Cablin Aqueous Extract (PCAE) Induces Mitotic Delay by Downregulating CDC2/Cyclin B1 in TNBC Cells (MDA‐MB‐231)

**DOI:** 10.1155/bri/4127073

**Published:** 2026-09-22

**Authors:** Kun-Ming Rau, Kuan-Ting Lee, Yung-Shun Su, Yi-Chiang Hsu

**Affiliations:** ^1^ Department of Hematology Oncology, E-Da Cancer Hospital, I-Shou University, Kaohsiung 824, Taiwan, isu.edu.tw; ^2^ Graduate Institutes of Medicine, College of Medicine, Kaohsiung Medical University, Kaohsiung 807, Taiwan, kmu.edu.tw; ^3^ Department of Recreation and Sport Management, Tajen University, Pingtung 907, Taiwan, tajen.edu.tw; ^4^ Department of Dermatology, Kaohsiung Medical University Hospital, Kaohsiung 807, Taiwan, kmuh.org.tw; ^5^ Department of Health and Beauty, Shu-Zen Junior College of Medicine and Management, Kaohsiung 821, Taiwan, szmc.edu.tw; ^6^ School of Medicine, I-Shou University, Kaohsiung 824, Taiwan, isu.edu.tw

**Keywords:** cell cycle, mitotic delay, pogostemon cablin aqueous extract (PCAE), triple-negative breast cancer (TNBC)

## Abstract

Pogostemon cablin (PC) is a traditional medicinal herb with diverse pharmacological activities, including reported anticancer effects. However, its therapeutic potential in triple‐negative breast cancer (TNBC) many unknowns remain. In this article, we investigated the antiproliferative activity of PC aqueous extract (PCAE) in MDA‐MB‐231 TNBC cells and explored the factors responsible for its anticancer effects. The cytotoxicity was evaluated by the MTT method, whereas apoptosis, cell‐cycle distribution, intracellular reactive oxygen species (ROS), mitochondrial membrane potential (MMP), and mitotic progression were analyzed. The expression of *G*_2_/*M* regulatory proteins was examined by western blotting. PCAE significantly suppressed the growth of TNBC cells in a dosage‐ and time‐dependent manner without inducing significant apoptosis, mitochondrial membrane depolarization, or caspase‐3 activation. Instead, PCAE markedly increased *G*_2_/*M* phase accumulation and mitotic index, accompanied by modulation of CDC25C phosphorylation and downregulation of CDC2 and cyclin B1 expression. These molecular alterations indicate that PCAE disrupts mitotic progression through inhibition of the CDC25C–CDC2/cyclin B1 regulatory axis. Collectively, our findings demonstrate that PCAE inhibits TNBC cell growth primarily by inducing mitotic delay rather than apoptosis, providing new mechanistic insight into the antitumor activity of PC and a promising therapeutic candidate for TNBC.

## 1. Introduction

Among the molecularly defined forms of cancer, TNBC comprises approximately 15%–20% of patients and lacks the three clinically established receptors, ER, PR, and HER2 expression. The absence of these gene targets is accompanied by a particularly aggressive disease phenotype, characterized by accelerated tumor growth, early development of distant metastases, frequent relapse, and relatively poor patient survival [1, 2]. Breast cancer (BC) remains one of the causes of tumor‐related mortality and cancer‐associated deaths among women worldwide [3]. The majority of tumor‐associated deaths—over 90%—result from metastasis, a highly complex and multistep process in which the primary lesion invades the surrounding stroma, intravasates into blood vessels, and ultimately establishes secondary tumors at distant organs [4–6]. Despite considerable advances in systemic therapy, treatment options for TNBC remain largely dependent on chemotherapy because of the lack of validated molecular targets. Furthermore, intrinsic tumor heterogeneity, therapeutic resistance, and treatment‐related toxicities continue to limit clinical outcomes [7, 8].

The therapeutic challenges associated with conventional anticancer regimens, particularly treatment‐related toxicity and incomplete clinical responses, have stimulated the search for alternative therapeutic approaches [9, 10]. Complementary and alternative medicine (CAM), particularly the use of medicinal plants and plant‐derived bioactive compounds, has therefore received increasing scientific attention. These natural products may exert anticancer effects by simultaneously modulating multiple molecular and cellular pathways, while some have demonstrated favorable toxicity profiles compared with conventional cytotoxic agents [11]. Such characteristics have encouraged continued investigation of medicinal plants as novel sources with anticancer potential.

The pharmacological diversity of natural products provides a valuable foundation for the discovery of new anticancer agents. Unlike compounds designed to act on a single molecular target, many phytochemicals can affect multiple signaling networks that regulate tumor cell survival, proliferation, inflammation, oxidative homeostasis, and immune responses. Consequently, medicinal plants and their bioactive constituents have been investigated for the potential to interfere with cancer‐associated processes while maintaining comparatively favorable tolerability [12, 13]. These characteristics make plant‐derived products attractive candidates for further evaluation as complementary or alternative approaches to cancer therapy.

Pogostemon cablin (PC), widely used in traditional Asian medicine [14], has long been used in the treatment of various ailments. Pharmacological investigations have demonstrated that PC possesses diverse biological activities, including antioxidant, antimutagenic, and immunomodulatory effects [15]. In addition, it has been traditionally prescribed for the management of conditions such as diarrhea, headache, and fever [16–18]. In classical medical practices, PC has also been recognized for its expectorant effects and has been used to treat disorders such as apoplexy and syncope related to food or fluid retention [18].

More recently, PC was investigated as a complementary antitumor agent in several malignancies, including colon cancer [19], acute myeloid leukemia [20], and liver cancer [21], further underscoring its broad therapeutic potential in oncology. In our previous study, we demonstrated that pogostemon cablin aqueous extracts (PCAEs) inhibited the growth of endometrial cancer Ishikawa cells primarily through induction of mitochondrial‐mediated apoptosis [14]. However, whether PCAE exerts similar biological effects in TNBC remains unknown.

We investigated the antiproliferative activity of PCAE in MDA‐MB‐231 cells with particular emphasis on its effects on mitotic progression. Unlike our previous findings in endometrial cancer, we demonstrate that PCAE does not primarily induce apoptosis under the present experimental conditions but instead suppresses TNBC cell growth through mitotic delay and *G*_2_/*M* phase arrest. Furthermore, we explored the molecular mechanisms underlying these effects by examining the regulation of cyclin B1, CDC2, CDC25C phosphorylation, and the mitotic marker MPM‐2. The PCAE was a potential therapeutic agent for TNBC treatment.

## 2. Materials and Methods

### 2.1. Materials

PC was supplied by Rich Fountain International Corp. 3‐(4,5‐dimethylthiazol‐2‐yl)‐2,5‐diphenyltetrazolium bromide (MTT) was from Sigma‐Aldrich (St. Louis, MO, USA) and dimethyl sulfoxide (DMSO). Culture‐related reagents were obtained from Gibco BRL (Grand Island, NY, USA). Unless otherwise indicated, reagents used throughout the experiments were of analytical grade.

### 2.2. PCAEs Preparation

The PCAE was generated following the extraction procedure reported in our previous publication [15]. The concentrated material was then dried for 48 h, yielding approximately 1.5 g of PCAE. The resulting crude extract was retained for subsequent experiments.

### 2.3. Cell Culture

MDA‐MB‐231 cells (RRID: CVCL_0062 from ATCC USA). The cells were propagated in Leibovitz’s L‐15 medium with FBS 10% (v/v), L‐glutamine (2 mM), and sodium bicarbonate (1.5 g/L). Cultures were maintained under atmospheric air without supplemental CO_2_. Cells undergoing exponential growth were selected for the experiments described below.

### 2.4. Cell Proliferation Assay

The effects of PCAE on MDA‐MB‐231 were quantified by measuring MTT reduction. Cells were distributed at 3 × 10^3^ cells/well and allowed to attach overnight. PCAE was subsequently added at a series of concentrations, followed by incubation for 1, 2, or 3 days. At the end of each treatment period, MTT was added, and the cells were incubated for 4 h with MTT. After the reaction was finished, DMSO. Absorbance (540 nm) measured from wells containing medium without cells was used for background correction. Three independent experiments were conducted, and results are shown as mean ± SEM.

### 2.5. Measurement of Apoptosis

Apoptotic cell populations were characterized by labeling with propidium iodide (PI)/Annexin V‐FITC by following a previous publication [22]. Flow cytometric profiles were analyzed with WinMDI Version 2.9.

### 2.6. Caspase 3 Activity Assay

Caspase‐3 activation was assessed by intracellular staining with a FITC‐labeled antibody (BD Pharmingen, USA) specific for active caspase‐3. MDA‐MB‐231 cells were treated with PCAE and subsequently harvested for antibody staining. Fluorescence was measured using a flow cytometer (FACSCalibur).

### 2.7. Cell Cycle Analysis

The distribution of cell‐cycle phases was determined from cellular DNA content following PI staining, as described in a previous publication [22]. Results were analyzed by FACSCalibur with WinMDI Version 2.9.

### 2.8. Analysis of Mitochondrial Membrane Potential (MMP)

The MMP (ΔΨm) was examined using the JC‐1 fluorescent probe, as described in a previous publication [15]. JC‐1 fluorescence was measured by FACSCalibur, and fluorescence profiles were quantified with WinMDI Version 2.9.

### 2.9. Determination of Intracellular ROS

Intracellular reactive oxygen species (ROS) were measured with the oxidation‐sensitive fluorescent probe DCFH‐DA (Molecular Probes, Invitrogen), as described in a previous publication [23].

### 2.10. Confocal Microscopy

To visualize the mitotic marker MPM‐2, TNBC cells were grown on coverslips at a density of 2 × 10^6^ cells and treated with PCAE for 16 h. Cells were subsequently fixed with the primary MPM‐2 antibody (Millipore, UK) for 30 min. Following PBS washing, an FITC‐conjugated rabbit antimouse secondary antibody (1:50; Serotec, Oxford, UK) was applied for 1 h at room temperature under light‐protected conditions. Nuclear DNA was counterstained with DAPI‐containing mounting medium. The stained preparations were examined using a confocal microscope.

### 2.11. Determination of the Mitotic Index

Mitotic progression was quantified according to the cellular expression of MPM‐2. Following PCAE exposure, cells were harvested and fixed in 70% alcohol. After washing, the cells were resuspended in IFA‐Tx buffer and incubated with MPM‐2 antibody. Samples were washed and subsequently exposed to FITC‐conjugated secondary antibody for another 60 min in the dark. Following the antibody‐labeling steps, cells were stained with *P* *I* *f* *o* *r* 30 min. MPM‐2‐associated fluorescence was then determined by FACSCalibur and quantified using WinMDI Version 2.9.

### 2.12. Western Blot Assay

For immunoblotting, cellular protein extracts were prepared, and 50–75 μg of total protein was loaded for electrophoresis on 12% SDS‐PAGE. Proteins were subsequently electrotransferred onto PVDF membranes and blocked with Odyssey blocking buffer; membranes were incubated with primary antibodies recognizing *β*‐actin, phosphorylated CDC25C, total CDC25C, CDC2, or cyclin B1. Following removal of unbound primary antibody by washing, membranes were incubated with IRDye‐conjugated secondary antibodies (LI‐COR Biosciences, USA) diluted 1:20,000. Immunoreactive bands were detected using the Odyssey infrared imaging system. Densitometric analysis was performed with Odyssey Software Version 2.1.

### 2.13. Statistical Analysis

Independent experiments were repeated at least three times, and quantitative results are expressed as mean ± SEM. For comparisons between two experimental groups, Student’s *t*‐test was applied. When more than two groups were compared, one‐way ANOVA followed by an appropriate post hoc test was used. Statistical significance was defined as *p* < 0.05.

## 3. Results

### 3.1. PCAE Suppresses the Viability and Proliferative Capacity of TNBC Cells

**FIGURE 1:**
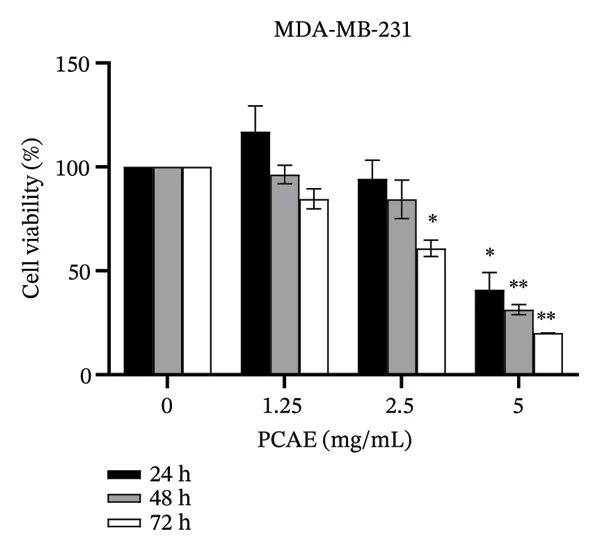
PCAE reduces MDA‐MB‐231 cell viability in a concentration‐ and exposure‐time‐dependent manner. The cells were incubated with PCAE at 0, 1.25, 2.5, or 5 mg/mL for 24, 48, or 72 h. Cell viability was subsequently determined by the MTT method and expressed relative to the untreated control. Statistical significance was evaluated using the *t*‐test. *p* < 0.05 was considered statistically significant compared with the untreated group (0 mg/mL PCAE). # vs. 24‐h group, whereas & vs. 48‐h group.

The cytotoxicity of PCAE was first examined in MDA‐MB‐231 cells using the MTT method. Cells were challenged with PCAE and maintained for 1–3 days. A progressive reduction in cell viability was observed as both the extract concentration and exposure period increased (Figure 1). Thus, PCAE exerted a concentration‐ and time‐dependent suppressive effect on the growth of MDA‐MB‐231 cells. These findings establish that PCAE effectively compromises the proliferative capacity of TNBC cells under the experimental conditions used in this study. Linear regression analysis further demonstrated a strong inverse relationship between PCAE concentration and cell survival (*y* = −26.346*x* + 132.25, *R* ^2^ = 0.9548). The calculated IC_50_ values are summarized in Table 1, indicating that approximately 50% growth inhibition was achieved within the tested concentration range. These findings demonstrate that PCAE effectively suppresses the proliferative capacity of MDA‐MB‐231 cells.

**TABLE 1:** The IC_50_ of PCAE in MDA‐MB‐231 cells.

Cell line	Tumor type	Drug (mg/mL)	24 h	48 h	72 h
Triple‐negative breast cancer (TNBC) cells					
MDA‐MB‐231	Human TNBC cell	PCAE	3.827	3.044	1.951
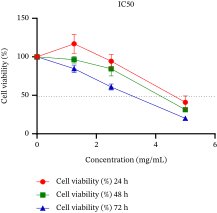					

*Note:* PCAE inhibited TNBC cell growth.

### 3.2. Growth Inhibition by PCAE Is Independent of Apoptosis and Necrosis

**FIGURE 2:**
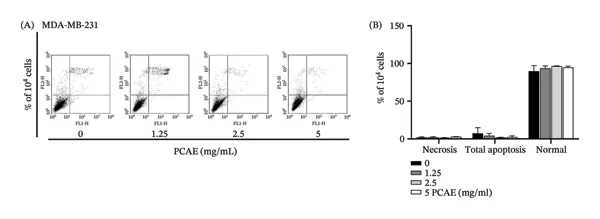
PCAE does not significantly increase apoptotic or necrotic cell populations in MDA‐MB‐231 cells. (A) Representative Annexin V‐FITC/PI flow cytometric profiles obtained following exposure of MDA‐MB‐231 cells to PCAE for 4 h. The distributions of viable, early apoptotic, late apoptotic, and necrotic cell populations were determined according to Annexin V and PI fluorescence.

Although PCAE markedly suppressed MDA‐MB‐231 cell proliferation, Annexin V‐FITC/PI staining demonstrated that this inhibitory effect was not accompanied by a significant increase in apoptotic or necrotic cell populations. Representative flow cytometric profiles are shown in Figure 2A, while quantitative analysis confirmed that neither early nor late apoptosis differed significantly from untreated controls (Figure 2B). Consistent with these observations, activation of caspase‐3 remained unchanged following 24 h of PCAE exposure. Collectively, these findings indicate that PCAE‐mediated growth inhibition occurs through an apoptosis‐independent mechanism.

### 3.3. PCAE Has Minimal Effects on MMP (ΔΨm)

**FIGURE 3:**
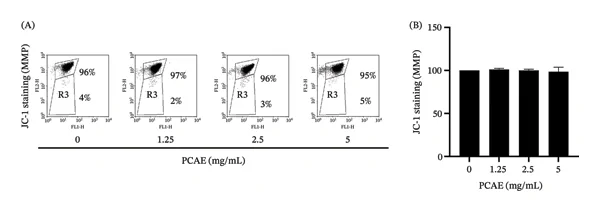
PCAE does not significantly alter mitochondrial membrane potential in MDA‐MB‐231 cells. (A) Representative flow cytometric profiles generated from JC‐1 staining after PCAE exposure. Red fluorescence represents JC‐1 aggregates associated with polarized mitochondria, whereas green fluorescence corresponds predominantly to the monomeric form of the dye. (B) Quantitative analysis of JC‐1 fluorescence showing the relative mitochondrial membrane potential following PCAE treatment. No statistically significant difference was detected between the PCAE‐treated and untreated groups.

Because alterations in ΔΨm are commonly associated with mitochondrial apoptotic signaling, we examined whether PCAE treatment affected mitochondrial function in cells. JC‐1 staining was used to monitor changes in mitochondrial polarization after 6 h of PCAE exposure. In untreated cells, JC‐1 fluorescence was predominantly detected in the red channel, consistent with the maintenance of mitochondrial membrane polarization (Figure 3A). PCAE treatment produced a modest shift toward green fluorescence; however, this change did not reach statistical significance. Quantitative analysis of the JC‐1 fluorescence profiles likewise revealed no significant difference in ΔΨm between PCAE‐treated and untreated cells (Figure 3B). These findings indicate that the growth‐inhibitory activity of PCAE is not accompanied by a significant loss of ΔΨm under the conditions examined.

### 3.4. PCAE Reduces Intracellular ROS Accumulation in MDA‐MB‐231 Cells

**FIGURE 4:**
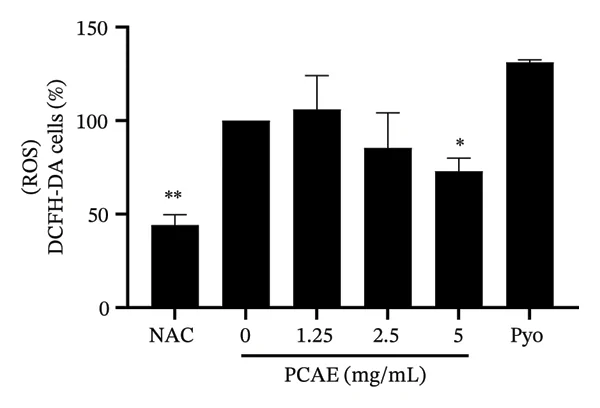
PCAE modulates intracellular ROS levels in MDA‐MB‐231 cells. MDA‐MB‐231 cells were exposed to PCAE (0, 1.25, 2.5, or 5 mg/mL) for 4 h, followed by DCFH‐DA staining and flow cytometric determination of intracellular ROS levels. NAC (10 mM) and pyocyanin (PYO; 200 mM) were used as ROS‐scavenging and ROS‐inducing controls, respectively. Representative flow cytometric profiles and quantitative fluorescence analyses are presented. Statistical significance was assessed using the *t*‐test, with *p* < 0.05 considered significant compared with the untreated control group.

To determine whether oxidative stress contributes to the growth‐inhibitory effect of PCAE, intracellular ROS levels were examined following PCAE exposure. MDA‐MB‐231 cells were treated with increasing concentrations of PCAE for 4 h and subsequently stained with DCFH‐DA. Flow cytometric analysis revealed a concentration‐associated reduction in intracellular ROS following PCAE treatment (Figure 4). Among the tested concentrations, 5 mg/mL PCAE produced a significant decrease in ROS levels relative to untreated cells. To further evaluate the relationship between PCAE treatment and intracellular ROS, ROS modulation was examined using N‐acetylcysteine (NAC) as an ROS scavenger and pyocyanin (PYO) as an ROS inducer. These treatments served as experimental controls for establishing the responsiveness of the ROS detection system. Overall, PCAE treatment did not increase intracellular ROS accumulation; instead, it was associated with reduced ROS levels in cells. Together with the lack of significant mitochondrial membrane depolarization and apoptosis observed in the preceding experiments, these findings suggest that excessive ROS generation is unlikely to be the primary mechanism underlying PCAE‐mediated growth inhibition.

### 3.5. PCAE Promotes *G*_2_/*M* Accumulation in MDA‐MB‐231 Cells

**FIGURE 5:**
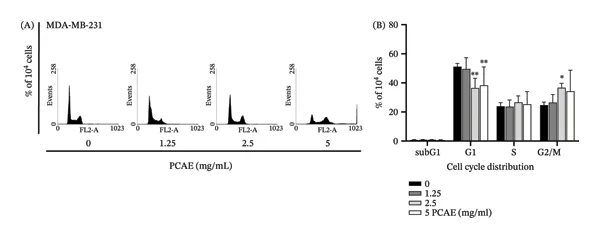
PCAE alters cell‐cycle distribution in MDA‐MB‐231 cells. (A) Representative flow cytometric profiles illustrating the distribution of cells across the *G*_0_/*G*_1_, S, and *G*_2_/*M* phases following a 24‐h exposure to PCAE. (B) Quantitative comparison of cell‐cycle populations showing an increase in the *G*_2_/*M* fraction accompanied by a decrease in the *G*_0_/*G*_1_ fraction with increasing PCAE concentrations. Statistical significance was determined using the *t*‐test. *p* < 0.05 versus the untreated control group (0 mg/mL PCAE).

Given that PCAE treatment did not significantly increase apoptotic cell populations, we next examined whether the reduction in cell growth was associated with alterations in cell‐cycle progression. TNBC cells were exposed to PCAE for 1 day, and DNA content was subsequently analyzed by flow cytometry. As shown in Figure 5A, PCAE treatment resulted in a distinct shift in cell‐cycle distribution. Increasing concentrations of PCAE were associated with a progressive expansion of the *G*_2_/*M* population, accompanied by a reduction in the proportion of cells in *G*_0_/*G*_1_. Quantitative analysis confirmed a positive linear relationship between PCAE concentration and the percentage of *G*_2_/*M*‐phase cells (*R* ^2^ = 0.8346), whereas the *G*_0_/*G*_1_ population showed an inverse relationship with PCAE concentration (*R* ^2^ = 0.9245) (Figure 5B). These findings indicate that PCAE interferes with normal cell‐cycle progression and promotes the accumulation of cell numbers at the *G*_2_/*M* transition.

### 3.6. PCAE Promotes Mitotic Accumulation in MDA‐MB‐231 Cells

**FIGURE 6:**
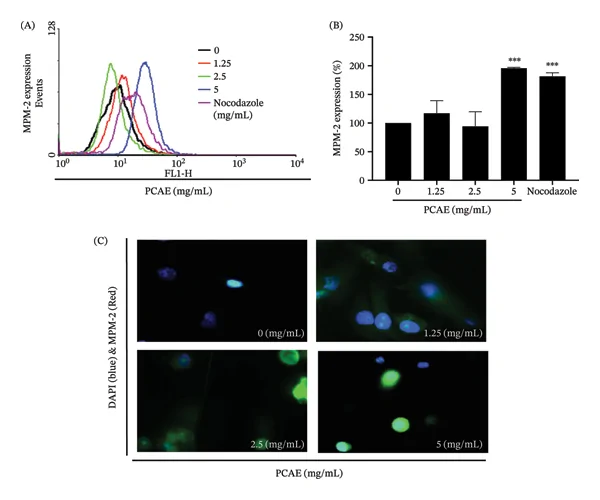
PCAE promotes mitotic progression‐associated MPM‐2 expression in MDA‐MB‐231 cells. (A) Representative confocal microscopy images showing MPM‐2 immunofluorescence following PCAE exposure. MDA‐MB‐231 cells were treated with PCAE for 24 h, followed by immunofluorescence staining for MPM‐2 (red). DAPI staining (blue) was used to visualize cellular nuclei. (B) Flow cytometric quantification of MPM‐2‐positive cells after 24 h of PCAE treatment. (C) The proportion of MPM‐2‐positive cells increased following PCAE exposure. Nocodazole (15 μg/mL), a microtubule‐disrupting agent that induces mitotic arrest, was included as a positive control. Quantitative data are presented as mean ± SEM from at least three independent experiments. *p* <  0.05 versus the untreated control group (0 mg/mL PCAE).

To determine whether *G*_2_/*M* accumulation reflected *G*_2_ arrest or mitotic arrest, mitotic progression was examined using the mitosis‐specific marker MPM‐2 (Figure 6A). MPM‐2 specifically recognizes phosphorylated epitopes that appear exclusively during mitosis, from early prophase to metaphase, and is therefore widely used as an indicator of mitotic progression and disturbance [24]. Nocodazole‐treated cells served as the positive control and exhibited the expected increase in MPM‐2‐positive cells (Figure 6B). Similarly, PCAE treatment markedly elevated MPM‐2 expression in a dose‐dependent manner, increasing the proportion of MPM‐2‐positive numbers from 27% in untreated cells to 97% at the highest PCAE concentration (Figure 6C). Interestingly, treatment with 2.5 mg/mL PCAE produced MPM‐2 expression comparable to that induced by nocodazole, suggesting that PCAE effectively disrupts mitotic progression. These findings support the conclusion that PCAE suppresses TNBC cell proliferation by inducing mitotic delay rather than merely causing *G*_2_ arrest.

### 3.7. PCAE Induces *G*_2_/*M* Phase Arrest in MDA‐MB‐231 Cells via Downregulation of CDC2 and Cyclin B1

**FIGURE 7:**
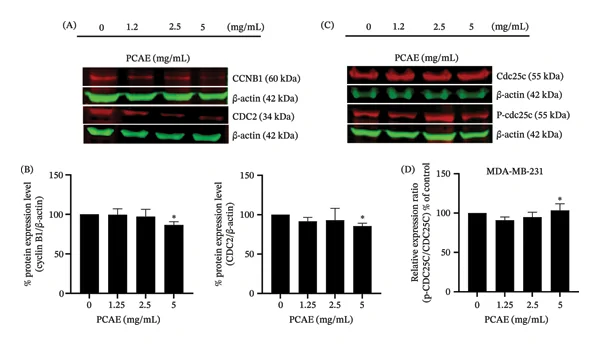
PCAE downregulates cyclin B1/CDC2 protein expression and induces phosphorylation of CDC25C in MDA‐MB‐231 cells. (A) Representative immunoblots showing the protein levels of CDC2/cyclin B1 following treatment with increasing concentrations of PCAE. (B) Densitometric quantification demonstrating concentration‐dependent changes in CDC2 and cyclin B1 expression. (C) Immunoblot detection of total CDC25C and phosphorylated CDC25C (p‐CDC25C) in PCAE‐treated cells. (D) Quantitative analysis of the p‐CDC25C/CDC25C ratio showing an increase in CDC25C phosphorylation following PCAE exposure. *p* < 0.05 is considered significant compared with the 0 mg/mL PCAE.

To elucidate the inhibitory molecular mechanisms of PCAE on MDA‐MB‐231 cell proliferation, first examined the expression of key regulatory proteins involved in the *G*_2_/*M* phase transition. Consistent with the observed *G*_2_/*M* accumulation, PCAE markedly reduced the expression of cyclin B1 and CDC2, two essential regulators of mitotic entry (Figure 7A). Densitometric analysis confirmed a concentration‐dependent decline in both proteins (Figure 7B), with significant suppression observed at 5 mg/mL PCAE. These molecular alterations are consistent with impaired activation of the CDC2/cyclin B1 complex and provide mechanistic evidence that PCAE‐induced mitotic delay is mediated through disruption of the *G*_2_/*M* regulatory machinery.

### 3.8. PCAE Alters CDC25C Phosphorylation in MDA‐MB‐231 Cells

To further characterize the molecular mechanism underlying PCAE‐induced *G*_2_/*M* phase accumulation, we next examined the phosphorylation status of CDC25C, a critical regulator of mitotic entry that controls activation of the cyclin B1/CDC2 complex [25]. Western blot analysis demonstrated that PCAE treatment altered the phosphorylation pattern of CDC25C in MDA‐MB‐231 cells (Figure 7C). Densitometric analysis revealed a concentration‐dependent increase in the *p*‐CDC25C/CDC25C ratio following PCAE exposure (*y* = 3.6965*x* + 97.28, *R* ^2^ = 0.765), with the greatest increase observed at the highest PCAE concentration (Figure 7D). Together with the concomitant reduction in CDC2 and cyclin B1 expression, these findings suggest that PCAE disrupts *G*_2_/*M* checkpoint regulation by modulating the CDC25C–CDC2/cyclin B1 signaling axis, thereby contributing to impaired mitotic progression in MDA‐MB‐231 cells.

## 4. Discussion

PC has long been utilized for the various tumors treatment in traditional Asian medicine, and numerous studies have sought to elucidate the mechanisms underlying its antitumor therapy [18, 26]. In the present study, we showed that PCAE exerts potent anticancer effects against MDA‐MB‐231 cells, primarily through the induction of mitotic delay and cell cycle arrest. Our findings provide experimental evidence that PCAE suppresses the proliferation of TNBC cells in vitro.

Mechanistically, PCAE treatment resulted in the downregulation of CDC2/cyclin B1 protein expression, accompanied by increased phosphorylation of CDC25C. CDC25C, a highly conserved dual‐specificity phosphatase, regulates the *G*_2_/*M* transition by dephosphorylating CDC2, thereby activating the CDC2/cyclin B complex [27]. The activated CDC2/cyclin B complex, in turn, phosphorylates CDC25C to enhance its phosphatase activity, forming a positive feedback loop that drives mitotic entry [27, 28]. Disruption of this regulatory axis has been reported to promote tumorigenesis by facilitating uncontrolled mitotic progression [29, 30].

Our findings suggest that the PCAE‐induced mitotic delay in MDA‐MB‐231 cells is not primarily mediated by CDC25C phosphorylation, but rather through the cyclin B1 and CDC2 downregulation. The suppression of these key *G*_2_/*M* regulatory proteins may contribute to irreversible growth arrest in cancer cells, underscoring the potential of PCAE as a modulator of cell cycle progression in TNBC.

Overall, our results provide the first evidence that PCAE effectively suppresses the growth of TNBC cells by delaying mitotic entry through inhibition of the cyclin B1/CDC2 axis. Given the aggressive nature and limited treatment options for TNBC, PCAE may serve as a promising candidate for development as a chemopreventive or adjunctive anticancer agent.

However, some limitations of this study should be acknowledged. First, all experiments were conducted in vitro using the MDA‐MB‐231 cell line, and thus the effects observed may not fully recapitulate PCAE’s activity within the complex in vivo tumor microenvironment. Second, the precise bioactive compounds responsible for the anticancer effects of PCAE remain unidentified, and potential synergistic or antagonistic interactions among its phytochemical constituents have yet to be clarified. Third, although downregulation of cyclin B1 and CDC2 appears to underlie PCAE‐induced mitotic delay, other molecular pathways may also contribute and warrant further exploration. The selected treatment durations were based on the different biological processes evaluated in this study. Cell cycle regulation and mitotic progression can occur earlier after exposure to anticancer agents, whereas apoptosis‐associated events, including MMP disruption and caspase activation, may require longer exposure periods. Therefore, the absence of significant apoptosis or mitochondrial dysfunction at the selected time points does not exclude the possibility of delayed apoptotic responses induced by PCAE.

Future investigations should focus on validating these findings in animal models to assess PCAE’s efficacy, pharmacokinetics, and safety in vivo [31, 32]. Bioassay‐guided fractionation and metabolomic analyses will be essential to pinpoint the active constituents and their molecular targets. Moreover, evaluating the potential synergistic effects of PCAE in combination with standard chemotherapeutics or targeted therapies could provide insight into its application as a complementary treatment for TNBC. Ultimately, clinical studies will be necessary to determine whether PCAE can be translated into a safe and effective option for BC prevention or therapy.

Furthermore, the current study evaluated apoptosis‐related events at specific time points; therefore, delayed apoptotic responses following prolonged PCAE exposure cannot be excluded. Future time‐course studies using extended treatment durations are necessary to fully characterize the temporal effects of PCAE.

## 5. Conclusions

In conclusion, this study demonstrates that PCAE exhibits potent anticancer activity against TNBC cells by inducing mitotic delay through inhibition of the cyclin B1/CDC2 regulatory pathway. These results provide mechanistic insight into the capacity of PCAE to suppress TNBC cell proliferation and highlight its potential as a complementary or preventive strategy in BC management. While the anticancer properties of PCAE and related phytochemicals are increasingly recognized, further studies—including in vivo validation, characterization of bioactive constituents, and clinical evaluation—are necessary to fully establish their therapeutic efficacy and translational potential.

## Author Contributions

Conceived the study and provided the funding: Kun‐Ming Rau and Yi‐Chiang Hsu. Performed the experiments and analyzed data: Kuan‐Ting Lee and Yung‐Shun Su. Conducted statistical analysis: Kuan‐Ting Lee. Wrote the manuscript: Kuan‐Ting Lee. Modified the manuscript: Kun‐Ming Rau and Yi‐Chiang Hsu. Supervision: Yi‐Chiang Hsu.

## Funding

This study was supported by the I‐Shou University, ISU‐109‐IUC‐01, ISU‐111‐IUC‐08, ISU‐114‐01‐09A; E‐Da Cancer Hospital, EDCHP111003, EDCHP112005, EDCHP112014, EDCHP113004, EDCHP113005, EDCHP114007, EDCHP115001.

## Disclosure

All authors read and approved the final manuscript.

## Conflicts of Interest

The authors declare no conflicts of interest.

## Data Availability

The data that support the findings of this study are available from the corresponding author upon reasonable request.
